# Supplementary material for: Molecular Implications of ADIPOQ, GAS5, GATA4, and YAP1 Methylation in Triple-Negative Breast Cancer Prognosis
Source: Int J Mol Sci. 2025 Nov 1;26(21):10652. doi: 10.3390/ijms262110652 (PMC12609348; doi:10.3390/ijms262110652)
Supplement: Supplementary file 1 [file ijms-26-10652-s001.zip › ijms-3919742-supplementary.pdf]

Supplementary Materials:

Table S1. Summary results of TCGA TNBC cohort in silico analysis (multivariable—expression).

		**Multivariable analyses**	
	mut.HR.%95CI	mut.pvalue	coef
		**Expression (high vs. low)**	
		OS	
ADIPOQ	1.03:(0.94-1.13)	0.509	0.0316
GAS5	1.1:(0.793-1.53)	0.564	0.0968
GATA4	0.891:(0.674-1.18)	0.417	-0.116
YAP1	0.776:(0.47-1.28)	0.324	-0.253
		PFI	
ADIPOQ	1.02:(0.931-1.12)	0.674	0.0195
GAS5	1.07:(0.777-1.46)	0.692	0.0639
GATA4	0.884:(0.68-1.15)	0.359	-0.123
YAP1	0.843:(0.527-1.35)	0.475	-0.171
		DSS	
ADIPOQ	1.05:(0.935-1.18)	0.411	0.0483
GAS5	1.18:(0.802-1.74)	0.4	0.166
GATA4	0.832:(0.572-1.21)	0.337	-0.184
YAP1	0.795:(0.441-1.43)	0.444	-0.23
		DFI	
ADIPOQ	1.03:(0.923-1.15)	0.589	0.0306
GAS5	1.21:(0.827-1.78)	0.321	0.194
GATA4	0.938:(0.706-1.25)	0.657	-0.0644
YAP1	0.921:(0.505-1.68)	0.788	-0.0822
		RFS	
ADIPOQ	1.01:(0.901-1.12)	0.914	0.0061
GAS5	1.32:(0.906-1.91)	0.149	0.274
GATA4	0.98:(0.738-1.3)	0.889	-0.0203
YAP1	0.987:(0.543-1.79)	0.965	-0.0133

OS—overall survival, DSS—disease-specific survival, DFI—disease-free interval, PFI—progression-free interval, RFS—relapse-free survival; *p*-values ≤ 0.05.

Table S2. Summary results of TCGA TNBC cohort in silico analysis (multivariable—methylation).

		**Multivariable analyses**	
	mut.HR.%95CI	mut.pvalue	coef
		**Methylation (high vs. low)**	
		OS	
ADIPOQ	0.0634:(0.00031-13)	0.31	-2.76
GAS5	133000:(0.054-3.29e+11)	0.116	11.8

GATA4	10.6:(0.196-570)	0.246	2.36
YAP1	0.00168:(1.91e-10-14800)	0.434	-6.39
		PFI	
ADIPOQ	0.0967:(0.000681-13.7)	0.356	-2.34
GAS5	6410:(0.00547-7.52e+09)	0.219	8.77
GATA4	0.561:(0.0089-35.3)	0.784	-0.578
YAP1	8.5e-06:(4.24e-12-17)	0.115	-11.7
DSS		DSS	
ADIPOQ	0.0361:(6.79e-05-19.2)	0.299	-3.32
GAS5	131000:(0.0108-1.6e+12)	0.157	11.8
GATA4	18.7:(0.155-2240)	0.231	2.93
YAP1	7.17e-06:(2.89e-14-1780)	0.23	-11.8
		DFI	
ADIPOQ	0.00681:(1.87e-05-2.48)	0.0973	-4.99
GAS5	3780:(3.1e-06-4.62e+12)	0.44	8.24
GATA4	1.97:(0.0216-179)	0.769	0.676
YAP1	4.76e-06:(2.17e-13-104)	0.155	-12.3
		RFS	
ADIPOQ	0.0158:(3.4e-05-7.38)	0.186	-4.15
GAS5	29200000:(0.347-2.46e+15)	0.0648	17.2
GATA4	3.21:(0.0252-408)	0.637	1.17
YAP1	5.91e-09:(8.38e-17-0.417)	0.0399	-18.9

OS—overall survival, DSS—disease-specific survival, DFI—disease-free interval, PFI—progression-free interval, RFS—relapse-free survival; *p*-values ≤ 0.05.
